# Acrylonitrile and Pullulan Based Nanofiber Mats as Easily Accessible Scaffolds for 3D Skin Cell Models Containing Primary Cells

**DOI:** 10.3390/cells11030445

**Published:** 2022-01-27

**Authors:** Markus Rimann, Astrid Jüngel, Sara Mousavi, Nicole Moeschlin, Maurizio Calcagni, Karin Wuertz-Kozak, Florian Brunner, Stefan Dudli, Oliver Distler, Christian Adlhart

**Affiliations:** 1 Institute of Chemistry and Biotechnology (ICBT), ZHAW Zurich University of Applied Sciences, 8820 Wädenswil, Switzerland; msv_sr@yahoo.com (S.M.); nicole.moeschlin@hotmail.com (N.M.); 2 Center of Experimental Rheumatology, University Hospital Zurich and Balgrist University Hospital, University of Zurich, 8091 Zurich, Switzerland; astrid.juengel@usz.ch (A.J.); stefan.dudli@usz.ch (S.D.); Oliver.Distler@usz.ch (O.D.); 3 Department of Plastic Surgery and Hand Surgery, University Hospital Zurich, University of Zurich, 8091 Zurich, Switzerland; Maurizio.Calcagni@usz.ch; 4 Department of Biomedical Engineering, Rochester Institute of Technology (RIT), Rochester, NY 14623, USA; kwbme@rit.edu; 5 Schön Clinic Munich Harlaching, Spine Center, Academic Teaching Hospital and Spine Research Institute of the Paracelsus Medical University Salzburg (Austria), 81547 Munich, Germany; 6 Department of Physical Medicine and Rheumatology, Balgrist University Hospital, University of Zurich, 8008 Zurich, Switzerland; florian.brunner@balgrist.ch

**Keywords:** 3D cell culture, microenvironment, tissue engineering, biomaterial, alternative methods

## Abstract

(1) Background: Three-dimensional (3D) collagen I-based skin models are commonly used in drug development and substance testing but have major drawbacks such as batch-to-batch variations and ethical concerns. Recently, synthetic nanofibrous scaffolds created by electrospinning have received increasing interest as potential alternatives due to their morphological similarities to native collagen fibrils in size and orientation. The overall objective of this proof-of-concept study was to demonstrate the suitability of two synthetic polymers in creating electrospun scaffolds for 3D skin cell models. (2) Methods: Electrospun nanofiber mats were produced with (i) poly(acrylonitrile-co-methyl acrylate) (P(AN-MA)) and (ii) a blend of pullulan (Pul), poly(vinyl alcohol) (PVA) and poly(acrylic acid) (PAA) (Pul/PVA/PAA) and characterized by scanning electron microscopy (SEM) and diffuse reflectance infrared Fourier transform (DRIFT) spectra. Primary skin fibroblasts and keratinocytes were seeded onto the nanofiber mats and analyzed for phenotypic characteristics (phalloidin staining), viability (Presto Blue HS assay), proliferation (Ki-67 staining), distribution (H/E staining), responsiveness to biological stimuli (qPCR), and formation of skin-like structures (H/E staining). (3) Results: P(AN-MA) mats were more loosely packed than the Pul/PVA/PAA mats, concomitant with larger fiber diameter (340 nm ± 120 nm vs. 250 nm ± 120 nm, *p* < 0.0001). After sterilization and exposure to cell culture media for 28 days, P(AN-MA) mats showed significant adsorption of fetal calf serum (FCS) from the media into the fibers (DRIFT spectra) and increased fiber diameter (590 nm ± 290 nm, *p* < 0.0001). Skin fibroblasts were viable over time on both nanofiber mats, but suitable cell infiltration only occurred in the P(AN-MA) nanofiber mats. On P(AN-MA) mats, fibroblasts showed their characteristic spindle-like shape, produced a dermis-like structure, and responded well to TGFβ stimulation, with a significant increase in the mRNA expression of *PAI1*, *COL1A1*, and *αSMA* (all *p* < 0.05). Primary keratinocytes seeded on top of the dermis equivalent proliferated and formed a stratified epidermis-like structure. (4) Conclusion: P(AN-MA) and Pul/PVA/PAA are both biocompatible materials suitable for nanofiber mat production. P(AN-MA) mats hold greater potential as future 3D skin models due to enhanced cell compatibility (i.e., adsorption of FCS proteins), cell infiltration (i.e., increased pore size due to swelling behavior), and cell phenotype preservation. Thus, our proof-of-concept study shows an easy and robust process of producing electrospun scaffolds for 3D skin cell models made of P(AN-MA) nanofibers without the need for bioactive molecule attachments.

## 1. Introduction

It has been widely accepted that cells grown in three dimensions (3D) represent the native tissue much better than cells cultured in standard monolayer conditions because they experience similar physiological cues, such as cell-cell/extracellular matrix (ECM) interactions, biochemical and mechanical signals, and nutrient and oxygen gradients [1]. This has a direct effect on biological properties including cellular morphology, proliferation, migration, cellular signaling, differentiation, and gene and protein expression [2,3].

One of the most prominent examples of 3D cell cultures includes in vitro human skin models. A driver for the development of skin equivalents has been the animal ban for cosmetics and ingredients testing in Europe effective since 2013. As a result, in vitro 3D human skin models have been further developed and have become robust tools for different applications not only in the cosmetics industry but also in academic research and R&D laboratories in the pharmaceutical industry [4,5,6].

The standard material for skin tissue engineering is collagen I, an animal tissue-derived biomaterial that exhibits natural cell compatibility and provides relevant biochemical and mechanical cues for mammalian cell growth since it represents the main proteinaceous component of the native ECM. To create 3D skin models, fibroblasts are first grown in the material to rebuild the dermal part of the skin [7], followed by seeding with keratinocytes to produce a stratified epidermis in an air-liquid interface culture [8,9,10]. Although being the most common biomaterial used in 3D skin models, collagen I has major drawbacks. As a natural product, it is prone to batch-to-batch variations. Furthermore, for ethical reasons, animal-derived products are less tolerated by society. Thus, new synthetic (bio-) polymers are being developed that aim to circumvent these limitations.

Furthermore, new technologies that enable the production of ECM-like structures in a controllable process, such as electrospinning, also support the development of alternative and improved 3D skin models. Electrospinning of polymers creates nanofibers that show morphological similarities to native collagen fibrils in size and orientation, albeit with relatively small pore size [11]. Both synthetic polymers such as acrylic polymers and biopolymers such as polysaccharides are interesting candidates for nanofiber ECM analogues of the human skin by providing suitable porosity, mechanical strength, and diffusion of nutrients [12].

Poly(acrylonitrile-co-methyl acrylate) (P(AN-MA)) belongs to the family of synthetic poly(1-acrylonitrile) (PAN) polymers. The presence of the methyl acrylate comonomer improves the plastic properties of PAN. These materials are generally considered non-toxic [13] while being lightweight and showing high strength, and resistance to corrosion and fatigue [14]. The polymers are used in various domains like medication, paints, electrical insulators, and artificial organs [15,16].

The second studied nanofiber ECM analogue was a blend of the three polymers Pullulan (Pul), poly(vinyl alcohol) (PVA), and poly(acrylic acid) (PAA), termed Pul/PVA/PAA. Pul is a natural biodegradable extracellular polysaccharide produced by the fungus *Aureobasidium pullulans* [17,18]. It has been described as non-toxic, non-immunogenetic, and exhibits anti-oxidative properties [19,20,21,22]. The biosynthetic polymer PVA exhibits hydrophilic, biodegradable, and biocompatible properties. Besides the biomedical field, it is also applied in the cosmetics, food, and pharmaceutical industries [23,24]. The third polymer PAA is added for crosslinking the Pul/PVA nanofibers. PAA is non-toxic and biologically inert, and its mechanical properties can easily be adjusted. The three polymers were mixed prior to electrospinning. The blending of various polymers is a useful method to enhance or modify the physicochemical characteristics of polymeric materials [25]. Blending is also well established to improve the properties of polymer solutions during the electrospinning process [26,27,28].

The aim of this proof-of-concept study is the generation of skin-like structures with the key primary skin cell types (dermal fibroblasts and keratinocytes) using electrospun nanofiber mats without specific pre-treatments to enhance cell attachment.

## 2. Materials and Methods

Poly(acrylonitrile-co-methyl acrylate) (P(AN-MA)) nanofiber mats: The general procedure of preparing nanofiber mats using high throughput free liquid surface electrospinning was described previously [26,27,28,29]. To prepare a 10 wt% (P(AN-MA)) solution, P(AN-MA) (Mw = 150,000 g mol^−^^1^, acrylonitrile = 91.5%, Haihang Industry Co. Ltd., Hainan, China) was dissolved in N,N-dimethylformamide (DMF, ≥99%, Merck, Buchs, Switzerland) for 24 h under mechanical stirring at room temperature (RT) until a homogeneous solution was obtained. The viscosity and electrical conductivity of the resulting solution were 840.5 mPas and 104.2 µS cm^−^^1^, respectively. The solution was electrospun on a NanoSpiderTM NS Lab 500 S (Elmarco s.r.o., Liberec, Czech Republic) using a wire electrode with a rotational speed of 1.5 rpm onto a moving paper substrate (5 mm min^−1^) at 62 kV with a collector distance of 22 cm, temperature ϑ = 25 °C, relative humidity RH = 39%.

Pullulan/poly(vinyl alcohol)/poly(acrylic acid) (Pul/PVA/PAA) nanofiber mats: To prepare a Pul/PVA/PAA solution, 200 g Pul solution (10 wt% in water, food-grade, Hayashibara Co. Ltd., Okayama, Japan) were mixed with 300 g PVA solution (10 wt% in water, Mw = 89,000–98,000 Da, DH = 99%, Merck, Switzerland) followed by adding 22 g of poly(acrylic acid sodium salt) (Mw = 5100 g mol^−^^1^, Merck, Switzerland) resulting in 4.2 wt% PAA with a Pul:PVA ratio of 4:6. The solution was electrospun on a NanoSpiderTM NS Lab 500 S using a drum electrode with a rotational speed of 2.0 rpm onto a moving paper substrate (10 mm min^-1^) at 70 kV with a collector distance of 20 cm, temperature ϑ = 27 °C, relative humidity RH = 26%. After electrospinning, the nanofiber mat was thermally cross-linked in air at 180 °C for 45 min [28].

Scanning electron microscopy (SEM) and fiber diameter measurement: For SEM visualization, the nanofiber mats were mounted on SEM specimen holders and sputtered with gold for 30 s at 20 mA with a sputter coater (Quorum Q150RS, Laughton, UK). Images were recorded using a Quanta 250 FEG (Thermo Fisher Scientific Inc., Waltham, MA, USA) with an accelerating voltage of 5 kV and a spot size of 2.5 at a working distance of 10 mm. From those images, nanofiber diameters were determined manually by measuring >100 fibers using the open-source image processing software ImageJ 1.50i.

Diffuse reflectance infrared Fourier transform (DRIFT) spectra: DRIFT spectra were recorded on a Bruker Tensor 37 (Bruker, Leipzig, Germany) Fourier transform infrared (FTIR) spectrometer using a Minidiff Plus DRIFT accessory (Specac Ltd., Orpington, UK). The resolution was set to 4 cm^−^^1^ and spectra were averaged for 128 scans. Reflectance signals were transformed according to Kubelka Munk (KM).

Preparation and sterilization of the membranes for cell culture experiments: P(AN-MA) and Pul/PVA/PAA electrospun nanofiber mats were (i) cut to 1 × 1 cm^2^ pieces and clipped into a 3D printed nylon clamp (custom-made transwell) or (ii) punched out by a biopsy puncher (discs of 5 mm). Prior to cell seeding, nanofiber mats were sterilized by incubation in ethanol (EtOH, 80% in PBS) for 2 h at RT, washed 3 times with PBS, and exposed further to UV light for 1 h. Subsequently, samples were pre-incubated with DMEM cell culture medium (Dulbecco’s Modified Eagle Medium (DMEM)/F12 medium, LifeTechnologies/Thermo Fisher Scientific, Basel, Switzerland) supplemented with 10% fetal calf serum (FCS) (heat-inactivated 56 °C for 20 min, Life Technologies/Thermo Fisher Scientific, Switzerland), 1% penicillin/streptomycin (P/S), 1% L-glutamine, 1% HEPES buffer and 0.2% Amphotericin B (all Thermo Fisher Scientific, Switzerland)—termed FB culture medium—for 24 h in an incubator at 37 °C and 5% CO_2_.

Human skin samples: All research on human-derived samples was conducted in compliance with the Declaration of Helsinki. The approval of the local ethics committee was obtained for the Department of Rheumatology; University Hospital Zurich and Balgrist University Hospital (approved ethics application 2017-01298 and 2017-00349). All participants signed an informed consent. Skin biopsies were obtained from donors undergoing surgery for non-infectious, non-inflammatory conditions (e.g., patients undergoing mamma reduction surgery at University Hospital Zurich) without evidence for systemic diseases and were considered to be healthy control (HC) skin (Table 1).

Isolation of primary human skin fibroblasts and keratinocytes: Primary human dermal fibroblasts (skin FBs) were obtained by outgrowth culture, and cells from passages 4 through 10 were used under standard culture conditions as previously described [30]. To induce fibrosis, cells were incubated with recombinant human transforming growth factor beta (TGFβ, 10 ng mL^−^^1^, PeproTech, London, UK). Primary human keratinocytes were isolated from skin biopsies of healthy individuals. The epidermis was removed mechanically after overnight incubation with dispase (10 µg mL^−^^1^ in PBS at 4 °C, Thermo Fisher Scientific, Basel, Switzerland). To isolate keratinocytes, the epidermis was incubated in 5-fold concentrated Trypsin/EDTA (3× for 2 min, LifeTechnologies/Thermo Fisher Scientific, Basel, Switzerland). The collected cell suspension was passed through a cell strainer (70 μm pores, Falcon, LifeTechnologies/Thermo Fisher Scientific, Basel, Switzerland) and incubated in Keratinocyte Serum Free Medium (K-SFM, Thermo Fisher Scientific, Basel, Switzerland) supplemented with Epidermal Growth Factor (EGF, 2.5 µg, Thermo Fisher Scientific, Basel, Switzerland), Bovine Pituitary Extract (BPE, 25 mg, Thermo Fisher Scientific, Basel, Switzerland), 1% P/S and 1% L-glutamine on collagen I pre-coated (40 µL mL^−^^1^ PBS, rat-tail, Corning; 3.65 mg mL^−^^1^, Brunschwig, Basel, Switzerland 1:10 in culture medium, 2 h at 4 °C) cell culture flasks. Cells were further cultured without collagen pre-coating and used from passage 2–4 for the experiments.

**Figure 1:**
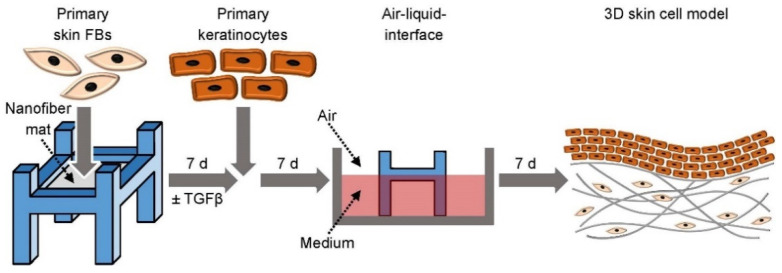
Experimental settings and schedule for skin cell model development: Seeding procedure of primary human skin FBs with or without primary human keratinocytes on electrospun nanofiber mats.

Seeding of primary skin FBs onto the nanofiber mats: Skin FBs were trypsinized, centrifuged, and resuspended in FB culture medium to a concentration of 1,000,000 cells mL^−^^1^ and 200,000 cells in 200 µL were seeded dropwise on the custom-made transwell with the nanofiber mats. The cell-laden nanofiber mats were then kept at 37 °C in the incubator for at least 30 min to promote cellular adhesion before adding FB culture medium. After 1 h, a second 200 µL aliquot of cell suspension was seeded on the other side of the nanofiber mats. The nanofiber mats in the custom-made transwell were cultured in a 12 well plate and the medium was changed every 2–3 days (Figure 1).

Metabolic activity: Punch biopsies (5 mm) of the electrospun nanofiber mats were placed in ultra-low attachment 96 well plates (Corning, Brunschwig, Basel, Switzerland) and seeded with 50,000 cells (skin FBs) in 50 µL cell culture medium 4 days before testing. The metabolic activity and proliferation of attached primary skin FBs on electrospun nanofiber mats was determined by the fluorometric Presto Blue HS assay (LifeTechnologies/Thermo Fisher Scientific, Basel, Switzerland) according to the manufacturer’s instructions on day 3, 7, and 14 after cell seeding. As controls, electrospun nanofiber mats without cells were used. The reagent was added for 4 h at 37 °C and 5% CO_2_. Measurements of fluorescence excitation and emission at 560 nm and 590 nm were obtained by transferring 100 µL aliquots of each specimen in triplicates (including the negative control consisting of culture medium and reagent) to 96 well plates. The mean of three scaffolds per condition was examined (three measurements per sample), and values with more than 20% variance of the same condition were excluded. Fluorescence intensities were determined by subtracting the negative control from the specimen readings (relative fluorescence units, RFU).

3D skin cell cultures with primary skin FBs and keratinocytes: Skin FBs in FB culture medium were seeded dropwise on the nanofiber mats (both sides) fixed in the custom-made transwells. After 7 days, isolated primary keratinocytes (200,000 cells per nanofiber mat in 200 µL, passage 2–4) in keratinocyte culture medium were seeded dropwise onto the nanofiber mats. To allow the cells to adhere, they were kept for 30 min at RT before adding keratinocyte culture medium. After 7 days, the stratification of the epidermis-like structure was started by air-liquid interphase cultures for additional 7 days according to established skin tissue engineering protocols [31]. For the culture of keratinocytes at the air-liquid interface, the Rheinwald Green (RWG) medium was used. RWG medium consists of three parts of DMEM and GlutaMAX-I (Thermo Fisher Scientific, Basel, Switzerland) and one part of F12 Nutrient Mixture (Ham’s F12, Thermo Fisher Scientific, Basel, Switzerland) supplemented with 5 µg mL^−^^1^ insulin, 0.4 µg mL^−^^1^ hydrocortisone, 0.18 mM adenine (all Thermo Fisher Scientific, Basel, Switzerland), 2 nM triiodothyronine (Sigma, Buchs, Switzerland), 0.1 nM choleratoxin, 5 µg mL^−^^1^ gentamycin, and 10 ng mL^−^^1^ EGF (all LifeTechnologies/Thermo Fisher Scientific, Basel, Switzerland) [32]. On day 21, the 3D skin cell constructs were removed from the clamps and used for RNA isolation and histological analysis (Figure 1).

TGFβ stimulation and gene expression analysis: Primary skin FBs (*n* = 3, passage 4–8) were cultured for 3 days after seeding onto electrospun nanofiber mats to allow the cells to adhere and to remove non-adherent cells by media change. Cells were starved in FB culture medium containing only 1% FCS for 24 h and stimulated with TGFβ (10 ng mL^−^^1^, in DMEM 10% FCS) for 48 h before the cells were lysed for RNA isolation. For control purposes, primary skin FBs (*n* = 5, passage 4–8) were additionally seeded in 6 well plates (300,000 cells per well) and treated identically. Total RNA was extracted using the miRNeasy kit (Qiagen, Hombrechtikon, Switzerland) inclusive DNase treatment according to the manufacturer’s instructions. Membranes were washed with PBS and lysed in Qiazol buffer (Qiagen, Hombrechtikon, Switzerland). RNA concentration was determined by spectrophotometer (Nanodrop, Thermo Fisher Scientific, Basel, Switzerland). cDNA was generated from 100 ng of RNA using the Sensifast cDNA synthesis kit (Labgene Scientific, Chatel-Saint-Denis, Switzerland) according to the manufacturer’s instructions. Resulting cDNA (20 µL) was diluted 1:10 with RNase-free water (Qiagen, Switzerland) and stored at −20 °C. Gene products were analyzed by qPCR, using Bio-Sensifast SYBR Green Master Mix (Labgene Scientific, Switzerland) and specific oligonucleotides in a MIC real-time PCR machine. Gene expression of plasminogen activator inhibitor-1 (*PAI1*), collagen I (*COL1A1*), and actin alpha 2 (*ACTA2*, alpha smooth muscle actin [*αSMA*]) was measured by quantitative real-time PCR and quantified using the ΔΔCq method with *GAPDH* as a reference gene. Each experiment was performed in duplicate. Specific qPCR oligonucleotide sequences are available in Table 2.

Phalloidin-TRITC staining of cells cultured on nanofiber mats: Cells on nanofiber mats were fixed in ice-cold methanol at −20 °C for 10 min and washed 3× in PBS. The samples were treated with 0.1% Triton X-100 (Sigma, Buchs, Switzerland 2 × 5 min) followed by a blocking step of 10% FCS in PBS for 45 min at RT and incubated with Phalloidin tetramethyl-rhodamine-isothiocyanate (TRITC) 1% DMSO (20 µg mL^−^^1^, Sigma, Buchs, Switzerland) for 1 h at RT in PBS for actin filament staining. After washing with PBS, nuclei were stained with Hoechst (1:2000, 5 ng mL^−^^1^, Roche, Basel, Switzerland), washed with PBS and water, and covered with fluorescence mounting medium (Dako, Hamburg, Germany).

Hematoxylin/Eosin—(H/E) and Ki-67-staining of FFPE slides: To visualize the cross-sections of the 3D skin cell models, formalin-fixed, paraffin-embedded (FFPE) 4 μm sections were stained with H/E according to standard protocol. The membranes were imaged using a confocal microscope (Nikon Eclipse Ti2, 10× and 20× magnification, Nikon, Amsterdam, The Netherlands) and a fluorescence microscope (Zeiss Apoptome.2, 10× magnification, Zeiss, Feldbach, Switzerland). After deparaffinization, sections were boiled for 10 min in sodium citrate buffer (10 mM, pH 6.0) for antigen retrieval followed by a treatment with 3% H_2_O_2_ in methanol for 15 min at RT to block endogenous peroxidase activity. Sections were incubated with anti-Ki-67 (rabbit-anti-human, Abcam, Cambridge, UK,) antibodies for 60 min at RT followed by an incubation (45 min) with the secondary antibodies (goat-anti-rabbit-HRP, Dako, Hamburg, Germany). Immunoreactivity was developed using 3-amino-9-ethylcarbazole (AEC kit, Abcam, Cambridge, UK) as the chromogen. Sections were finally counterstained with Mayer’s hematoxylin, washed in PBS, and mounted in an aqueous mounting medium (Dako, Hamburg, Germany). The membranes were imaged using a confocal microscope (Nikon Eclipse Ti2, 10× and 20× magnification).

Statistical analysis: All continuous variables were expressed as the mean ± standard error of the mean. Results were analyzed by a non-parametric Kruskal Wallis test using MATLAB (version 3.6.1) and differences were considered statistically significant if *p* < 0.05, denoted as “*”.

## 3. Results

### 3.1. Characterization of the Nanofiber Mats

**Figure 2:**
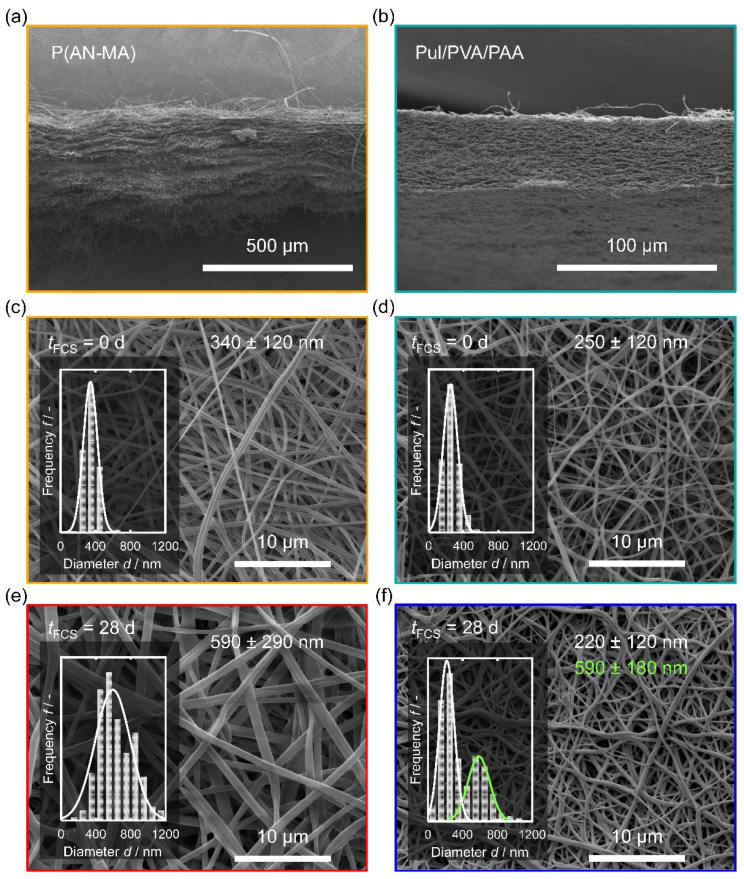
SEM images of P(AN-MA) and Pul/PVA/PAA nanofiber mats showing cross-sections (**a**,**b**) and top views before (**c**,**d**) and after 28 days of culture conditions in FCS (**e**,**f**). The insets in (**c**–**f**) show histograms of the nanofiber diameters. Inset (**f**) shows a bimodal fiber distribution after 28 days of incubation caused by attaching fibers (green).

P(AN-MA) and Pul/PVA/PAA nanofiber mats used for the cell culture were obtained through electrospinning. To facilitate electrospinning of Pul and the cross-linking agent PAA, the water-soluble sacrificial polymer PVA was added. Figure 2 shows the morphology of the P(AN-MA) and Pul/PVA/PAA nanofiber mats.

Both materials were successfully electrospun providing defect-free nanofibers with narrow fiber diameter distributions (340 ± 120 nm, P(AN-MA), Figure 2c, and 250 ± 120 nm, Pul/PVA/PAA, Figure 2d). SEM images of cross-sections showed a significant difference between P(AN-MA) and Pul/PVA/PAA. While Pul/PVA/PAA is a porous single block material of approx. 40 µm thickness (Figure 2b), the P(AN-MA) scaffold consisted of multiple layers of thinner nanofiber mats which delaminated during the preparation of the cross-sections (Figure 2a).

To probe the behavior of the nanofiber mats during culture conditions, mats were sterilized and incubated in DMEM with 10% FCS for 28 d analogous to the in vitro cultures. Both materials retained their fibrous structure (Figure 2e,f), but significant changes were observed. P(AN-MA) fibers showed significant swelling and almost doubled their diameter to 590 ± 290 nm. This could either be due to water acting as a porogen, swelling due to intercalation of FCS components, or due to coating with FCS. The SEM image (Figure 2e) revealed no pores and if water had been the swelling agent, we would expect bone-like fiber cross-sections after SEM sample preparation during high vacuum. Coatings usually leave residues of coating materials in inter fiber pores [33] even if the coating was homogeneous, therefore diffusion of FCS components into the P(AN-MA) matrix may explain the swollen fibers. For Pul/PVA/PAA on the contrary, other morphological changes were observed after 28 d in FCS (Figure 2f). At many places, the fibers were touching each other at their full length, meaning that the fibers must have become flexible in aqueous FCS and cohesion forces have pulled them together while immersed in FCS or during drying. In addition, the material became less porous. Fiber diameters were now showing a bimodal distribution due to touching fibers.

**Figure 3:**
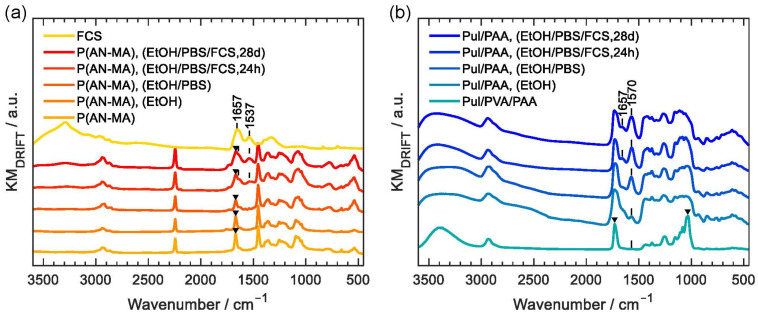
DRIFT infrared spectra of P(AN-MA) (**a**) and Pul/PVA/PAA (**b**) nanofiber mats at different treatment steps.

Figure 3a shows DRIFT infrared spectra of P(AN-MA) nanofiber mats at different stages of treatment prior to cell seeding. Electrospun nanofibers P(AN-MA) showed all the characteristic peaks of P(AN-MA), in particular the distinctive nitrile (C≡N) vibration at 2243 cm^−1^ and the CH_2_ bending vibration at 1454 cm^−1^. The characteristic peaks at 1734 cm^−1^ (marked with a triangle) can be associated with the (C=O) stretching vibration of the methacrylic acid functional groups [34].

We investigated to which extent sterilization would affect the P(AN-MA) nanofibers, since sterilization may alter the properties of electrospun nanofibers [35]. After washing with 80% EtOH for 2 h (P(AN-MA), (EtOH)) and after the following washing with PBS buffer (P(AN-MA), (EtOH/PBS)) the characteristic methacrylic acid peaks remained. Before cell seeding, the sterilized samples were placed in culture medium for 24 h (P(AN-MA), (EtOH/PBS/FCS, 24 h)). This resulted in two additional peaks at 1537 cm^−1^ and 1657 cm^−1^ (shoulder). These two peaks had the characteristic wavenumbers of amide II (combined N-H bending and C-N stretching of the –CO-NH– group) and the amide I (C=O) stretching vibration, which indicates partial adsorption of FCS components to the P(AN-MA) nanofiber skeleton. Such adsorption of FCS components could be beneficial for the attachment of skin FBs on the fiber surface. Cells were cultured for up to 28 days and P(AN-MA) nanofibers incorporated even more FCS during the cultivation period, as seen from the increased peaks at 1537 cm^−1^ and 1657 cm^−1^ (P(AN-MA), (EtOH/PBS/FCS, 28 d)). The spatial resolution of FTIR does not allow to differentiate between surface adsorption of FCS or intercalation in between the P(AN-MA) polymer chains, but SEM images of the thickened fibers, Figure 2e, were free of any bridging or pore filling structures at the fiber interfaces, which may be expected in case of surface deposition.

Pul/PVA/PAA nanofiber mats were also investigated with DRIFT infrared, Figure 3b. A significant change after washing with 80% EtOH was observed since two peaks at 1730 cm^−1^ and 1034 cm^−1^ are missing (Pul/PVA/PAA, (EtOH)). These peaks are characteristic for PVA, namely the (C=O) stretching vibration of acetate groups from partially hydrolyzed poly(vinyl acetate) [36] and the (C-O) stretching vibrations of PVA. This indicates that the water-soluble sacrificial PVA had been removed during EtOH sterilization. After washing with PBS, the peak at 1570 cm^−1^ became more intense (Pul/PAA, (EtOH/PBS)). This is due to stretching vibrations of carboxylate groups (C=O) that are generated by the deprotonation of carboxylic acid groups. In turn, the characteristic broad band O-H stretching vibration between 3300 and 2500 cm^−1^ [37] of the free carboxylic acid group is depleting. After treatment with FCS for 24 h (Pul/PAA, (EtOH/PBS/FCS, 24 h)) and 28 d respectively (Pul/PAA, (EtOH/PBS/FCS, 28 d)), an additional peak at 1657 cm^−1^ indicated the presence of FCS components (amide I). The amide II vibrations were hidden by the strong C=O stretching vibration at 1570 cm^−1^. The characteristic FCS peaks in the Pul/PAA scaffolds were less pronounced than in the P(AN-MA) scaffolds, which means that less FCS was adsorbed. This is consistent with the SEM observations, where significant swelling was only observed in the case of P(AN-MA). A possible reason could be the thermal crosslinking reaction, which is required to render Pul/PAA water stable. The resulting chemically cross-linked polymer network will impede the intercalation through individual FCS components while the van der Waals bound segmented P(AN-MA) polymer chains could facilitate the uptake of FCS components into the intra polymer chain space like plasticizers.

### 3.2. Viability of Human Skin FBs on Nanofiber Mats

**Figure 4:**
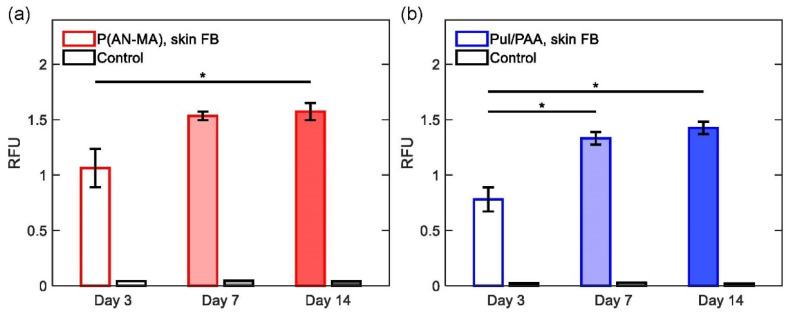
Metabolic assay of skin FBs (*n* = 3) growing on electrospun nanofiber mats for 14 days analyzed by PrestoBlue HS Cell Viability Assay and control (electrospun nanofiber mats without cells). The mean of the fluorescence intensity shown as RFU of three scaffolds per condition with three measurements per sample were examined. Significant proliferation was found from day 3 to 14 on P(AN-MA) (**a**) and from day 3 to 7 and 14 for Pul/PAA (**b**). Significance *p* < 0.05 (*).

As a measure of cell proliferation, metabolic activity of skin FBs seeded on P(AN-MA) and Pul/PAA (*n* = 3 each) nanofiber mats were assessed over 14 days of culture. On both nanofiber mats P(AN-MA) and Pul/PAA, skin FBs showed significant proliferation from day 3 to 14 (both *p* = 0.0495, non-parametric Kruskal Wallis), Figure 4.

### 3.3. Reaction to Pro-Fibrotic Stimuli

**Figure 5:**
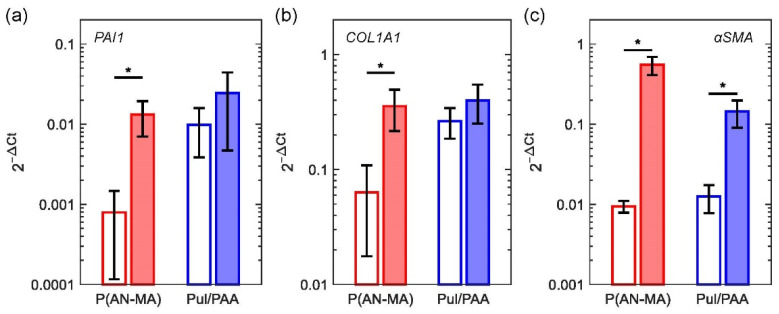
Gene expression of (**a**) *PAI1*, (**b**) *COL1A1* and (**c**) *ACTA2* (*αSMA*) analyzed by qPCR of human skin FBs cultured on electrospun nanofiber mats P(AN-MA) and Pul/PAA after 48 h TGFβ (10 ng mL^−1^) stimulation, white bar = without stimulation and filled bar = TGFβ stimulation, *n* = 3. Significance *p* < 0.05 (*).

Skin FBs grown in standard monolayer cultures (*n* = 5) showed significantly upregulated gene expression of plasminogen activator inhibitor 1 (*PAI1*)/*SERPINE1*, *ACTA2* (*αSMA*), and collagen I (*COL1A1*), as expected. Cells grown on P(AN-MA) electrospun nanofiber mats (*n* = 3) showed the same significant upregulation of gene expression (all *p* = 0.0495), whereas cells grown on Pul/PAA electrospun nanofiber mats showed a trend but not a significant induction of these pro-fibrotic genes, Figure 5a–c. Since skin FBs on Pul/PAA nanofiber mats were less sensitive to TGFβ stimulation (*PAI1*, *p* = 0.827, *COL1A1*, *p* = 0.513, *αSMA*, *p* = 0.0495) we decided to continue with P(AN-MA) nanofiber mats where a significant upregulation of the three genes was observed.

### 3.4. Attachment and Morphology of Skin FBs and Keratinocytes on Electrospun Nanofiber Mats

**Figure 6:**
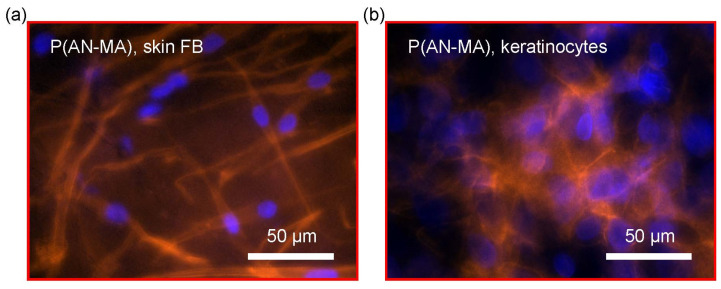
Cell distribution and morphology on electrospun nanofiber mats. Cells were cultured for 7 days on P(AN-MA) nanofiber mats and stained with Phalloidin-TRITC and Hoechst: (**a**) Primary skin FBs and (**b**) primary keratinocytes.

Phalloidin conjugated to TRITC is used to stain cellular F-actin, the main components of the cellular cytoskeleton. Staining primary skin FBs growing on electrospun nanofiber mats showed typical spindle-type shape and keratinocytes growing on electrospun membranes show typical polygonal appearance, on P(AN-MA) (Figure 6a,b).

### 3.5. 3D Skin Cell Model Using Electrospun Nanofiber Mats as a Scaffold

**Figure 7:**
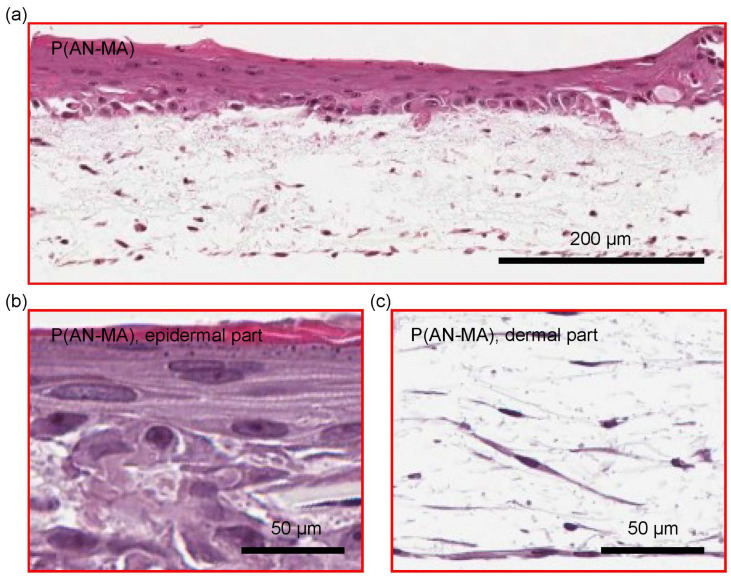
H/E staining of the 3D skin cell model with primary skin cells grown on P(AN-MA) electrospun nanofiber mats for 21 days showing skin FBs in the dermal part and keratinocytes building the epidermis-like structure. In (**a**) the entire skin cell model is shown including the epidermal and dermal part; (**b**,**c**) are close-up view of the epidermal and dermal part, respectively.

Primary skin FBs and keratinocytes grown in and on P(AN-MA) electrospun nanofiber mats built a skin-like structure containing dermis- and epidermis-like compartments as shown with H/E staining, Figure 7a. The typical stratification of the epidermis is shown in Figure 7b. Skin FBs with their characteristic spindle type structure were homogeneously distributed in the P(AN-MA) nanofiber mat and closely located to the overlaying skin keratinocytes, Figure 7c.

**Figure 8:**
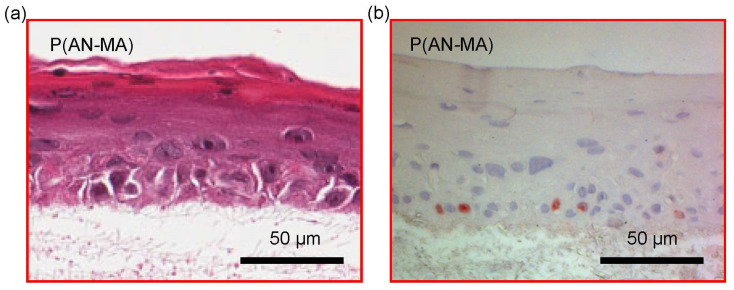
H/E and Ki-67 staining of the 3D skin cell model showing stratified epidermis-like structures and proliferating keratinocytes on P(AN-MA) electrospun nanofiber mats. (**a**) H/E staining, (**b**) Ki-67 staining of proliferating cells (red) in the epidermal part.

Primary keratinocytes cultured on top of skin FBs grown in P(AN-MA) nanofiber mats formed a stratified epidermal-like structure as shown with H/E staining in Figure 8a. In Figure 8b, sections of the epidermal part were stained with Ki-67 to visualize proliferative cells (red). Only in the lower epidermal layer Ki-67-positive cells were found (Figure 8b).

## 4. Discussion

3D cell culture models have received increasing interest in different research areas including basic, applied, and industrial research [38]. Among those models, skin models belong to the most advanced ones [39,40]. Most of the developed skin models are based on animal-derived collagen I, with several drawbacks such as batch-to-batch variability [41]. Consequently, the use of other materials, such as chitosan, polycaprolactone (PCL), and poly(ethylene glycol) (PEG) has been investigated [4]. To simulate the inherent ECM architecture of skin, with its nanofibrous structure and porosity, electrospinning of these polymers can be employed [42,43,44,45,46]. Despite the evident success of electrospun scaffolds, there are numerous ongoing challenges, such as achieving suitable cell infiltration [43], cell adhesion [47], or mechanical properties [43]. Research is mainly focused on biopolymers (e.g., hyaluronic acid, alginate, collagen, silk protein, fibrinogen, chitosan, starch, and poly(3-hydroxybutyrate-co-3-hydroxyvalerate (PHBV)) [48] or on synthetic polymers such as poly(lactic acid-coglycolic acid) (PLGA), PCL or PLA [42,46,48]. To promote cell adhesion, proliferation, migration, or morphology, bioactive molecules are commonly added to the scaffolds, in particular collagen, gelatin, or elastin [47], which are however associated with challenges in terms of batch-to-batch variability or adhesion efficiency.

Less attention has been drawn to the potential of synthetic or polysaccharide-based polymers such as acrylonitrile or pullulan without the incorporation of specific bioactive molecules. First reports indicate that, due to their pore size and fiber diameter, electrospun PAN nanofiber scaffolds are suitable in soft tissue regeneration [15,49], and specifically for growing fibroblast [50,51]. Pure PAN nanofibers were shown to allow high cell growth rates and additives, such as gelatin, can have a positive effect on skeletal muscle cells by e.g., improving cell differentiation [52,53]. Furthermore, aligned nanofibers from the copolymer P(AN-MA) were shown to promote fibronectin network formation [54]. While PAN or P(AN-MA) nanofiber scaffolds have not been tested for 3D skin models, Pul has already been used as a biomaterial for skin tissue engineering. When topically administered as a gel, skin tissue regeneration was accelerated by enhancing collagen synthesis and wound contraction in rats [55]. However, until now, only a few examples report the use of electrospun Pul blends e.g., with cellulose acetate for tissue engineering applications [56,57]. In our experiments, Pul was blended with PVA to enhance the physical properties for electrospinning [29]. The water-soluble PVA is commonly used as sacrificial polymer and was removed from the Pul/PVA/PAA nanofiber mats during EtOH sterilization, as confirmed by DRIFT data, Figure 3b, leaving the cross-linked Pul/PAA blend as the scaffold before cell seeding.

Both scaffolds, P(AN-MA) and Pul/PAA, were found to be cell compatible as demonstrated by the skin FBs cell viability and proliferation over time (Figure 4). Furthermore, fibroblasts and keratinocytes adhered to both scaffolds and exhibited spindle-like and polygonal appearance, respectively. However, differences between P(AN-MA) and Pul/PAA scaffolds were also observed.

Firstly, although Pul-based nanofibers can efficiently adsorb solutes [58], FCS adsorption was more pronounced on P(AN-MA) scaffold as indicated by the intensive amide I and amide II vibrational peaks (Figure 3). The reason could lie in the specific nature of the P(AN-MA) copolymer. The distorted acrylonitrile sequence through the presence of a methyl acrylate unit at roughly every tenth position [59] could weaken intra-chain interactions of otherwise crystalline acrylonitrile-only PAN nanofibers [60]. Pul/PAA on the other hand was thermally cross-linked to render Pul/PAA water stable. Cross-linking could impede massive intercalation of FCS components. FCS adsorption may have had a positive effect on cell adhesion to P(AN-MA).

Secondly, the SEM images of the cross-sections of P(AN-MA) and thermally cross-linked Pul/PVA/PAA, Figure 2a,b, show a significant difference in the morphology of these two scaffolds. The Pul/PVA/PAA nanofiber mat consists of a compact 3D network of cross-linked fibers with reinforced fiber-fiber junctions, whereas the cross-section of the P(AN-MA) nanofiber mat reveals a slaty structure with weak fiber-fiber interaction. Furthermore, swelling processes result in increased pore size in the P(AN-MA) scaffold, which could have facilitated infiltration of skin FBs.

Thirdly, TGFβ stimulation led to an up-regulation of the pro-fibrotic genes *PAI1*, *COL1A1*, and *αSMA* (Figure 5), which is an important and standard functionality test of skin FBs [61]. This effect was more pronounced with the P(AN-MA) scaffolds compared to Pul/PAA. Interestingly, the basal gene expression levels of *PAI1* and *COL1A1* of cells in Pul/PAA nanofiber mats was similar to TGFβ-treated samples and as high as the TGFβ-stimulated P(AN-MA) samples. This demonstrates a lack of TGFβ-responsiveness of cells in Pul/PAA nanofiber mats, which might not be an ideal material for the development of a skin model.

Our subsequent investigations on P(AN-MA) scaffolds demonstrated suitable skin FBs infiltration into the nanofiber network, with the generation of a dermal structure. Once populated with keratinocytes, formation of an epidermal-like structure was confirmed, which was similar to a previous study where we used a bioprinting approach [62].

In summary, we produced a P(AN-MA) nanofiber mat that is suitable for cell infiltration, supports the development of a dermis-like structure, and allows keratinocyte-induced formation of a stratified epidermal-like structure on top. The nanofiber mat is produced in a robust manner without the need of attaching cell adhesive bioactive components. Electrospun P(AN-MA) nanofiber scaffolds hold promise for skin model generation and are not limited to the development of models for healthy skin applications, e.g., barrier function [5], but they may be applied for diseased skin models, e.g., systemic sclerosis (SSC). Furthermore, other tissue engineering applications may benefit from the cell-adhesive and biocompatible properties of the material.

## Figures and Tables

**Table 1:** Patient characteristics. HC = healthy control, F = female.

Patient Information (Nr.)	State	Birth Year	Biopsy Year	Biopsy Location	Sex
1	HC	1969	2018	upper arm	F
2	HC	1971	2017	breast	F
3	HC	1969	2017	breast	F
4	HC	1970	2017	upper arm	F
5	HC	1958	2018	arm	F
6	HC	1957	2017	upper arm	F

**Table 2:** Primer sequences used for this study.

Gene of Interest	Forward Primer	Reverse Primer
*GAPDH* (reference)	5′-GGGAAGCTTGTCATCAATGGA-3′	5′-TCTCGCTCCTGGAAGATGGT-3′
*COL1A1*	5′-CCGATGGATTCCAGTTCGAG-3′	5′-GGTAGGTGATGTTCTGGGAG-3′
*αSMA*	5′-GAACATGGCATCATCACCAA-3′	5′-TGGTGCCAGATCTTTTCCAT-3′
*PAI1*	5′-GCTCAGACCAACAAGTTCAACT-3′	5′-CAATGAACATGCTGAGGGTGT-3′

## Data Availability

Data are available from M.R., A.J. and C.A. upon request.
